# Hepatic Hydrothorax—An Independent Decompensating Event Associated with Long-Term Mortality in Patients with Cirrhosis

**DOI:** 10.3390/jcm10163688

**Published:** 2021-08-20

**Authors:** Daniela Matei, Rares Craciun, Dana Crisan, Bogdan Procopet, Tudor Mocan, Sergiu Pasca, Roxana Zaharie, Bogdan Popovici, Zeno Sparchez

**Affiliations:** 1 Department of Internal Medicine, “Iuliu Hatieganu” University of Medicine and Pharmacy, 400012 Cluj-Napoca, Romania; dmatei68@gmail.com (D.M.); craciun.rares.calin@elearn.umfcluj.ro (R.C.); bogdan.procopet@umfcluj.ro (B.P.); mocan.tudor@umfcluj.ro (T.M.); pasca.sergiu123@gmail.com (S.P.); zahariedeliaroxana@gmail.com (R.Z.); zsparchez@yahoo.co.uk (Z.S.); 2 Regional Institute of Gastroenterology and Hepatology, 400162 Cluj-Napoca, Romania; 3 Department of Gastroenterology, Cluj-Napoca Municipal Hospital, 400139 Cluj-Napoca, Romania; 4 Department of Thoracic Surgery, “Leon Daniello” Pulmonology Hospital, 400371 Cluj-Napoca, Romania; bpopovici10@yahoo.com

**Keywords:** hepatic hydrothorax, pleural effusion, decompensated cirrhosis, mortality, portal hypertension

## Abstract

**Background:** Hepatic hydrothorax (HH) is an understudied complication of decompensated cirrhosis. We aimed to evaluate the long-term prognosis of patients with HH by comparing them with a matched non-HH group. **Methods:** This retrospective study included 763 consecutive patients hospitalized for decompensated cirrhosis and ascites. Ninety-seven patients with HH were matched for survival analysis with non-HH patients based on liver disease severity. **Results:** The prevalence of HH was 13.1%. Patients with HH had significantly worse overall liver function. Upon matching, patients with HH had a lower long-term survival (15.4% vs. 30.9% at 5 years) with a mean overall survival of 22.2 ± 2.2 months for the HH group vs. 27.1 ± 2.6 months for the non-HH group (Log Rank–0.05). On multivariate survival analysis using Cox regression, the MELD-Na score, ALBI grade, hepato-renal syndrome, and grade III ascites had a significant impact on mortality in patients with HH. In patients with HH, a MELD-Na score ≥ 16, ALBI grade III, hepato-renal syndrome, or severe ascites delineated high-mortality risk groups. **Conclusions:** HH is consistently associated with more advanced liver disease. Patients with HH have worse long-term survival, their prognosis being closely intertwined with overlapping decompensating events.

## 1. Introduction

### 1.1. Background

Hepatic hydrothorax (HH) is an important, albeit understudied decompensating event in cirrhosis. It is commonly defined as a transudative pleural effusion, typically exceeding 500 mL, in patients with chronic liver disease and portal hypertension, in the absence of underlying cardiopulmonary disease. The estimated prevalence of HH among decompensated cirrhotic patients is between 5% and 10% [1,2,3,4,5,6], with figures exceeding 20% being reported [7].

HH commonly occurs in patients with ascites. The most widely agreed upon pathophysiological pathway for developing HH is the formation of peritoneal–pleural communications through micro- and macroscopic diaphragmatic defects. These defects appear to be more frequent on the right side of the diaphragm, which is more fibrous and prone to collagen fiber deterioration, explaining the predominance of right-sided pleural effusions. Differences in absorptive properties between the pleura and the peritoneum, combined with different pressure environments (negative inspiratory pressure exerting a vacuum effect through the peritoneal–pleural communications) might account for a different response to diuretic treatment and the few cases of isolated HH in patients with no ascites [1,2,3,4,5,6].

While HH typically occurs in end-stage liver disease, little is known about the specific prognostic impact of HH. Furthermore, its long-term influence on mortality, as well as the natural history of this complication and the interrelation between HH and the other, more established and well-defined decompensating events remains mostly unknown.

### 1.2. Aims

The main objective of our study was to evaluate the long-term survival of patients with HH, compared to a propensity-matched non-HH cohort.

As secondary objectives of our research, we tried to determine the basic profile of a patient with HH when compared to non-HH patients with decompensated cirrhosis, based on liver disease severity expressed by clinical and laboratory data, conventional prognostic systems, and accompanying decompensating events. Furthermore, we aimed to evaluate the impact of coexisting decompensating events during the index presentation on disease course and long-term mortality.

## 2. Materials and Methods

### 2.1. Study Design

This was a single-center retrospective study. A total of 763 consecutive patients with cirrhosis and ascites admitted in a tertiary care facility, over an 18-month timespan (January 2012—August 2013) were included. The institutional ethics committee approved the study. Written informed consent was obtained from all patients.

Hepatic hydrothorax was diagnosed based on the presence of pleural effusion in the absence of other associated cardiopulmonary conditions. The presence of pleural effusion was diagnosed using pleural ultrasound, chest X-ray, or CT scan. All patients had either a conventional chest X-ray or a thoracic CT scan to account for underlying lung disease (pneumonia, tumors, other lesions), as well screen for cardiomegaly (suggestive of a potential overlap with heart failure). When heart failure was suspected on clinical grounds, laboratory (NT-proBNP), imaging or electrocardiogram, a full cardiology check-up was performed, including cardiac ultrasonography. Thus, patients with underlying heart failure, lung disease, malignancies, autoimmune conditions, or other established causes of pleural effusion were excluded. Patients with HH lost to follow-up or who had undergone TIPS placement or liver transplantation during follow-up were also excluded (*n* = 3).

Our initial cohort was split into two groups according to the presence or absence of HH for the comparison of demographic, clinical, and biological data. Subsequently, a propensity-score 1:1 matching was performed based on disease staging (MELD-Na score, Child–Pugh class, age) for survival and natural history analysis.

### 2.2. Variables and Data Collection

All relevant clinical and biological data, including complete patient history, were collected on the first admission. Decompensated cirrhosis and the decompensating events were defined according to the latest EASL Clinical Practice Guidelines on Decompensated Cirrhosis [8]. Overt encephalopathy was defined as hepatic encephalopathy grade 2 to 4, according to the West Haven criteria [9]. Ascites was classified according to the most recent position paper published by the International Ascites Club [10]. 

All the laboratory work-up was performed on an automatic analyzer (Konelab 30 I-Thermo Electron Corp, Helsinki, Finland).

#### 2.2.1. Imaging

All patients were screened for ascites and pleural effusion on admission using ultrasonography. Furthermore, all patients had either a standard chest X-ray or a thoracic CT scan to screen for underlying pulmonary disease (pneumonia, tumors, other lesions). Patients in which heart failure was suspected on clinical, biological (NT-proBNP), or ECG grounds had a trans-thoracic heart ultrasonography evaluation to exclude decompensated cardiac disease.

#### 2.2.2. Prognostic Scores

The Child–Pugh score was calculated according to the original article published by Pugh et al. in 1973 [11]. The MELD-Na score was calculated according to Biggins et al. [12]. The ALBI score and grading system was implemented as described by Johnson et al. [13].

#### 2.2.3. Follow-Up

After the initial hospitalization and the study inclusion, all patients were followed up either in our ward or in the initial referring center. Therefore, data regarding decompensating events was only registered on the index presentation (prior decompensation history and concurrent decompensating events). Subsequent decompensating events were not included in our analysis due to high data heterogeneity, which would have contributed to significant bias. Data regarding mortality were obtained from national insurance registries, as inquired in August 2019. Data regarding TIPS placement and liver transplant were obtained from the specific registries. 

### 2.3. Statistical Analysis

Continuous variables with normal distribution were expressed as mean ± standard deviation (SD). The comparison was performed using the two-tailed Student’s *t*-test. The continuous variables with non-normal distribution were expressed as median and 95% confidence interval (CI). The comparison was performed using the Mann–Whitney U test. The chi-square test was used for categorical variables. Propensity score (PS) matching was performed at a 1:1 HH to the non-HH ratio to account for the disparity in disease staging between groups. The PS was calculated using a logistic regression model, including variables such as age, gender, cirrhosis etiology, MELD-Na, Child–Pugh score, and class. The Kaplan–Meyer curves with the log-rank test were used for survival analysis. The impact of different variables on survival time was assessed using the univariate Cox proportional hazards model. We estimated the contribution of each variable by the odds ratio (logistic regression) and hazard ratio (Cox) with its 95% confidence interval. All variables that had a significant influence on survival were included in a multivariate Cox proportional hazards model. Model overfitting is a significant challenge in studying the prognosis of decompensated liver disease. The statistical design aimed to limit this issue: if a certain variable was already included in a predictive scoring system or intimately tied to a complication (i.e., serum creatinine and hepato-renal syndrome—HRS), the decision was made not to include it as a separate variable in multivariate analysis. However, if multiple scoring systems included the same variable (bilirubin in ALBI, MELD-Na and Child-Pugh), or are intimately tied to a decompensating event (MELD-Na and hepato-renal syndrome, which are both impacted by creatinine), the predictive systems or decompensating events were treated as independent prognostic constructs, as their value expands beyond isolated laboratory metrics. The threshold for statistical significance was set at *p* = 0.05. MedCalc 13.3.9.0 software and SPSS software version 15.0 (SPSS Inc., Chicago, IL, USA) were used for the statistical analysis.

## 3. Results

### 3.1. Characteristics of Patients with and without Hepatic Hydrothorax

Between January 2012 and August 2013, seven hundred and sixty-three consecutive patients were hospitalized for decompensated cirrhosis and were considered for inclusion in our study. Among them, one hundred (13.1%) had HH. All patients had their ascites sampled, and among the patients with HH, 61 (62.8%) had their pleural effusions tapped, confirming the transudative nature. Evacuation thoracocentesis was performed in the initial hospital stay in 35 (36.08%) patients. A complete comparison between groups based on the presence of HH is illustrated in Table 1. Patients with HH had worse ascites severity, more frequent overt hepatic encephalopathy (74% vs. 54.6% *p* < 0.001), HRS (9% vs. 3.16%, *p* = 0.005), and had a higher chance of developing acute-on-chronic liver failure (ACLF) during the initial hospital stay (10% vs. 2.1%, *p* < 0.001). Patients with HH had a significantly worse liver function expressed by lower serum albumin, total protein, and serum sodium levels and increased total bilirubin and INR. They were in a higher Child–Pugh class, had higher MELD-Na scores, and a higher ALBI grade.

### 3.2. Long-Term Survival of Patients with Hepatic Hydrothorax

To assess prognosis directly related to HH, the 97 eligible patients with HH were matched for MELD-Na, Child–Pugh score, and age with 97 non-HH patients. The patients were censored after 72 months, resulting in a median follow-up of 12 months (interquartile range—27.5 months) in the HH group and 15 months (interquartile range—29 months) for the control group. During the follow-up, 149 patients (76.8%) died: 82 (84.5%) in the HH group and 67 (69.1%) in the control group (*p* = 0.01). Upon matching, there were no significant discrepancies between groups regarding gender distribution, liver disease etiology, laboratory workup (bilirubin, albumin, INR), decompensation history or concomitant decompensating events.

Univariate survival analysis using the Cox proportional hazards model on the matched group (including both HH and non-HH patients) has shown a significant impact on mortality for HH, with a HR of 1.37 (95% CI: 1.00–1.89, *p* = 0.05), along with multiple other prognostic variables and decompensating events, as illustrated in Table 2. 

Hepatic hydrothorax, along with the MELD-Na score, ALBI grade, HRS, and grade III ascites retained their significance on multivariate analysis (Table 3). Considering the widely discussed strong association among spontaneous bacterial peritonitis, acute kidney injury, and hepato-renal syndrome, we decided to only include the latter in the multivariate analysis, given its wider prognostic implications.

**Figure 1:**
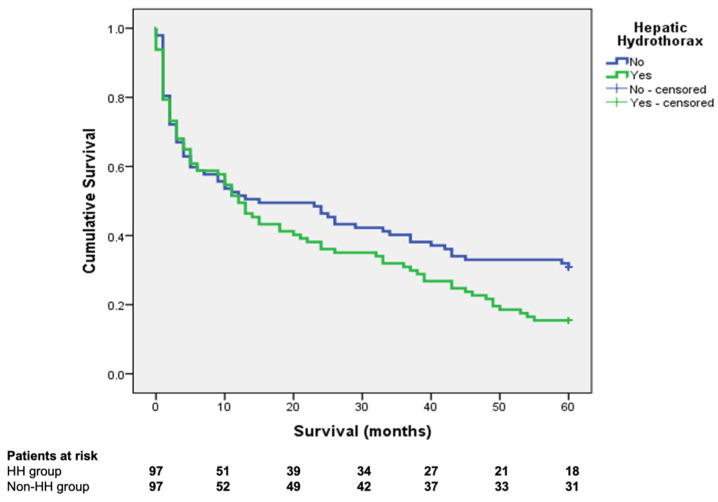
The Kaplan–Meier survival curves for the hepatic hydrothorax and control groups (log-rank 0.05).

The overall survival at 1, 2, and 5 years was 49.5%, 36.1%, and 15.4%, respectively, in the HH group and 51.5%, 46.4%, and 30.9%, respectively, in the control group (Log Rank—0.05) (Figure 1).

### 3.3. Natural History of HH and the Impact of Other Decompensating Events on Survival in Patients with HH

Univariate survival analysis using the Cox proportional hazards model in the HH group (Table 4) has shown that total bilirubin, kidney dysfunction, and HRS during the hospital stay, sodium levels, serum albumin, grade III ascites, SBP, MELD-Na, and ALBI score and ALBI grade were associated with survival. The same analysis was performed in the non-HH group. The variables associated with survival largely overlapped and followed the same trendline, except for serum albumin, which appeared to not influence survival. 

All included variables retained significance on multivariate analysis in the HH group (Table 5). Multivariate analysis of the control group, which included MELD-Na, ALBI grade, HRS, and grade III ascites revealed that MELD-Na (HR 1.01, 95% CI—1.00–1.05) and HRS (HR 3.25, 95% CI—1.05–11.57) were independently associated with survival.

**Figure 2:**
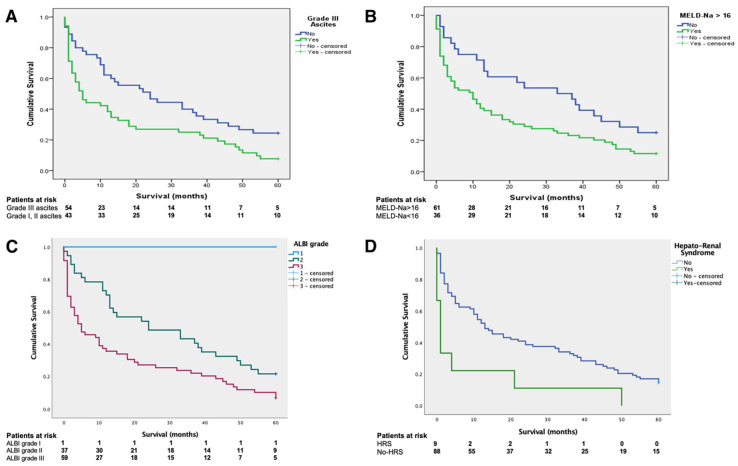
The subgroup survival analysis for patients with hepatic hydrothorax, based on the presence of grade III ascites (**A**), a MELD-Na score exceeding 16 (**B**), ALBI grade (**C**), and hepato-renal syndrome on the index presentation (**D**).

Based on these data, further subgroup analysis was performed on patients with HH (Figure 2). Consequently, patients with HH and grade III ascites had significantly worse survival when compared to patients with mild and moderate ascites, with a mean survival of 17.03 ± 2.88 months vs. 28.28 ± 3.38 months (log-rank < 0.01) (Figure 2A). Significantly different survival curves appeared when stratifying HH patients based on a MELD score of 16 (Figure 2B). Patients with scores exceeding this threshold had a mean survival of 18.56 ± 2.56 months, compared to 31.35 ± 4.27 for scores below 16 (log-rank 0.02). The survival gap increased along with the MELD-Na score, as exceeding a cut-off value of 20 has led to a mean survival of 12.88 ± 2.93 months, compared to 29.72 ± 2.98 months. Figure 2C depicts the survival curves based on ALBI grade, with the sole patient with ALBI grade 1 surviving the entire follow-up, patients with ALBI grade 2 (*n* = 37) having a mean survival of 26.56 ± 5.21 months, and ALBI grade 3 (*n* = 59) having a mean survival of 19.21 ± 3.21 months (log-rank 0.01). All patients with HH and HRS on the index presentation died during follow-up (*n* = 9), having a significantly worse survival (8.66 ± 5.53 months) compared to patients with no HRS, *n* = 88 (23.64 ± 2.41 months), as depicted in Figure 2D (log-rank 0.01).

## 4. Discussion

Our study has shown that patients with HH have a significantly higher long-term mortality rate when compared to patients with no HH, matched by liver function, in the absence of liver transplantation. Overall, patients with HH tend to be on the more severe end of the liver disease severity spectrum, as expressed by markers of liver function (bilirubin, INR, albumin, and sodium levels), decompensation profile (hepatic encephalopathy, hepato-renal syndrome, ACLF), and prognostic systems (MELD-Na, Child–Pugh, ALBI). When assessing the prognosis of patients with HH, clinical and biological factors (the severity of ascites, SBP, HRS, low serum albumin levels) appear to significantly influence mortality. The MELD-Na score and ALBI grade seem to be good mortality predictors for patients with HH; of note, the Child–Pugh class appears to be less reliable in predicting outcomes in this subgroup. Furthermore, patients with HH and a MELD-Na score above the threshold value of 16 or with severe ascites are significantly prone to a worse outcome, with the survival gap further increasing along with the MELD-Na score.

Some of the correlations, as mentioned above, represent common scientific knowledge and they need no further confirmation (i.e., the predictive values of MELD and Child–Pugh scores and the impact of albumin, bilirubin, kidney function, or ascites on overall prognosis) [7,10,13,14,15]. However, the novelty of our approach consists of emphasizing the role of HH as a predictive factor for long-term mortality. Therefore, screening patients with decompensated cirrhosis and ascites for HH to facilitate quicker access to more advanced treatment options or simply to ensure stricter discharge criteria and a tighter follow-up regimen might be worth studying from a cost/benefit standpoint. 

We compared our results with the few published papers available on this topic. The estimated prevalence for HH is in the range of 5% to 10% among cirrhotic patients with ascites [3,4,6]. A small prospective study on a Pakistani population reported a prevalence of 11.2% [16]. A retrospective study published by Badillo et al. reported a prevalence of 16% on a cohort of 495 patients [17]. Our results were in between these figures, at 13.1%.

Regarding short- and mid-term mortality of patients with decompensated cirrhosis and HH, data from prospective studies are scarce. One aforementioned study [17] reported 30-day, 90-day, and one-year mortality rates of 10%, 26%, and 57%, respectively. A large retrospective study conducted in Taiwan, which included 3487 cirrhotic patients with pleural effusions, reported 30-day, 90-day, 1-year, and 3-year mortality rates of 20.1%, 40.2%, 59.1%, and 75.9%, respectively [18]. However, in the latter study, data were collected using diagnostic and procedure codes from the electronic records, and it included only patients with pleural effusions requiring drainage, thus making an accurate diagnosis of hepatic hydrothorax without underlying or overlapping conditions rather difficult. Therefore, higher mortality rates might be explained by adding patients with more extensive collections (thus requiring drainage) and with more severe comorbidities (i.e., heart failure, pneumonia, empyema, or cancer).

The key contribution of our paper in the field of HH is the description of the long-term natural history of this complication in the absence of a radical therapeutic approach to portal hypertension such as TIPS placement or liver transplantation. As previously mentioned, mortality data extending beyond 3 years is lacking, and to our knowledge, no previously published reports comparing survival discrepancies on matched samples grouped by the presence or absence of HH are available. While certainly less impactful on the immediate prognosis when compared to other more established decompensating events (such as ascites, variceal bleeding, or kidney dysfunction), HH appears to enter the limelight later on in the timeline of disease progression, significantly altering prognosis. Additionally, by further stratifying HH patients based on their liver function, another prognostic dichotomy emerges, as patients with MELD scores exceeding 16 appear to have a significantly worse outcome. These data, combined with the fact that patients with HH appear to be in worse overall shape, might prove valuable in selecting patients for more expensive procedures, such as TIPS placement or, ultimately liver transplantation, in limited resources settings or in the decision to refer the patient to the closest tertiary care facility.

Another aspect worth discussing is the place of HH among the other decompensating events in cirrhosis. There are multiple cases in clinical practice in which HH requires a sophisticated therapeutic approach. However, HH is rarely the sole feature of a decompensated patient. Its occurrence is typically accompanied at least by ascites, and frequently, the overall homeostasis is disrupted on multiple levels. Therefore, more often than not, the critical therapeutic objectives are aimed towards treating the other complications, while HH is actively addressed when it becomes symptomatic. Diuretics, salt restriction, and therapeutic thoracentesis are currently recommended as the first-line treatments [2,8]. Other, more specific solutions such as chemical pleurodesis, indwelling pleural catheter drainage, or pleuro-venous shunts are rarely employed, with equivocal long-term results [19,20,21]. 

Furthermore, there are only a few reports in which TIPS placement or liver transplantation is performed for HH as the main indication. The most important contribution regarding TIPS in this niche was published back in 2001, in the twilight of the bare-metal stents era, on 40 patients [22]. While the initial response was acceptable, with a 71% complete HH resolution rate, stent dysfunction occurred in half of the cases, rendering a poor 35% one-year relapse-free survival. Most of the other available data come from a systematic review published in 2015 which, given the timeframe, also included a large percentage of TIPS with bare-metal stents, with a largely sub-optimal response when compared to other indications [23]. No long-term survival data is available. On the other hand, liver transplantation provides the definitive cure for HH, along with resolving the underlying liver disease. Available reports suggest that post-transplantation survival for patients with HH is similar to other indications, reaching up to 70% at 8 years [24,25]. Yet, given the scarcity of data with regards to waiting list mortality, up until this point, there was no evidence-based grounding for providing MELD exception points. Our research might provide valuable insight in this direction. Unfortunately, our country has relatively low liver transplantation rates, and TIPS was only introduced in our center as a routine procedure in late 2016, initially for repeated variceal hemorrhage, consequently explaining the low number of patients undergoing these procedures in our cohort of patients with advanced liver disease and multiple decompensating events. 

In this light, identifying high-risk groups among patients with HH appears to be key in categorizing patients in whom HH is an accompanying symptomatic feature of ascites or a hidden marker for a poor outcome. Thus, multiple clinical questions arise. As clinicians, should we treat it as an isolated complication, with local intervention and reoccurrence prevention? Or should we restrain from an invasive approach unless it provides symptomatic relief and treats the underlying liver disease as a whole? Further studies on HH could shed light on these issues. 

We are certainly aware of the limitations of our study. The single-center, retrospective design certainly impacts the value of our findings. Furthermore, not all the patients were followed up on in our department, and, therefore, details about the cause of death or other associated clinical events are lacking. In our design, we did not explicitly account for treatment, which of course, can be a significant prognostic modifier, nor for the HH-related symptom burden, which might have helped better understand the course of this decompensating event. Given the nature of our design, it was relatively difficult to account for salt restriction compliance and diuretic dose variation throughout follow-up. Nevertheless, we excluded patients who underwent TIPS or liver transplantation, which are major prognostic modifiers.

## 5. Conclusions

Hepatic hydrothorax is a frequently overlooked decompensating event in cirrhosis, closely interconnected with the other complications of portal hypertension, yet it is an independent entity. HH is associated with higher long-term mortality and, among patients with HH, those with high-grade ascites, HRS, a MELD-Na score over 16, or ALBI grade III have a significantly worse prognosis. 

## Figures and Tables

**Table 1:** Comparison between groups based on the presence or absence of hepatic hydrothorax.

Variable	No Hepatic Hydrothorax	Hepatic Hydrothorax	*p*-Value
N	663 (86.89)	100 (13.10)	
Gender—Female (N, %)	238 (35.80)	42 (42)	0.24
Age (years)	60.57 ± 10.86	59.38 ± 9.20	0.29
Liver disease etiology (N, %)			
Alcohol Use	279 (42.1)	44 (44)	0.60
Viral	256 (38.6)	41 (41)	
Mixed (viral + alcoholic)	66 (10)	5 (5)	
Other	62 (9.4)	10 (10)	
Serum albumin (g/dL)	3.14 ± 0.60	2.86 ± 0.54	<0.01
Total bilirubin (mg/dL) *	2.30 (2.10–2.40)	2.98 (2.58–3.41)	<0.01
INR *	1.54 (1.51–1.58)	1.67 (1.59–1.77)	<0.01
Child–Pugh A (N, %)	147 (22.17)	6 (6)	<0.01
Child–Pugh B (N, %)	290 (43.74)	40 (40)	
Child–Pugh C (N, %)	226 (34.08)	54 (54)	
MELD	16.08 ± 5.90	18.26 ± 6.18	<0.01
MELD-Na	17.02 ± 6.95	20.16 ± 7.40	<0.01
ALBI Score	−1.59 ± 0.63	−1.29 ± 0.61	<0.01
ALBI Grade 1 (N, %)	35 (5.27)	1(1)	<0.01
ALBI Grade 2 (N, %)	337 (50.8)	39 (39)	
ALBI Grade 3 (N, %)	251 (37.85)	60 (60)	
Serum creatinine (mg/dL) *	0.78 (0.75–0.80)	0.86 (0.75–0.95)	<0.01
30 days mortality (N, %)	99 (14.93)	19 (19)	0.15
6 months mortality (N, %)	184 (27.75)	32 (32)	0.08
Concomitant decompensating events			
Grade 1 ascites (N, %)	240 (36.19)	18 (18)	<0.01
Grade 2 ascites (N, %)	192 (28.95)	28 (28)	
Grade 3 ascites (N, %)	231 (33.63)	54 (54)	
Hepatic encephalopathy	362 (54.60)	74 (74)	<0.01
Hepato-renal syndrome (N, %)	30 (4.52)	9 (9)	<0.01
SPB OA (N, %)	64 (9.65)	21 (21)	<0.01
ACLF	14 (2.1)	10 (10)	<0.01
History of variceal bleeding (N, %)	130 (19.6)	23 (23)	0.43

Continuous variables are shown as follows: mean ± SD (standard deviation) for normally distributed variables or median * (95% CI—confidence interval), for skewed variables. HH—hepatic hydrothorax; INR—international normalized ratio; MELD—model for end-stage liver disease; ALBI—albumin/bilirubin; SPB OA—spontaneous bacterial peritonitis on admission; ACLF—acute-on-chronic liver failure during initial stay.

**Table 2:** Univariate Cox proportional hazards model in the entire matched cohort (*n* = 194 patients).

Variables	Hazard Ratio	95% Confidence Interval	*p*-Value
Age (years)	1.01	0.99–1.03	0.16
Gender (female)	0.65	0.45–0.92	0.01
Total bilirubin (mg/dL)	1.05	1.03–1.08	<0.01
INR on admission	1.1	0.90–1.60	0.21
Creatinine on admission (mg/dL)	1.05	0.89–1.23	0.55
Kidney dysfunction during hospital stay	3.27	1.80–5.95	<0.01
Hepato-renal syndrome during hospital stay	4.72	2.18–10.21	<0.01
Sodium levels (mEq/L)	0.93	0.91–0.96	<0.01
Serum albumin (g/dL)	0.54	0.40–0.74	<0.01
Grade III ascites	1.93	1.40–2.68	<0.01
Spontaneous bacterial peritonitis	2.26	1.50–3.39	<0.01
Hepatic encephalopathy	1.23	0.87–1.73	0.23
Child–Pugh Class C	1.24	0.79–1.66	0.41
MELD score	1.06	1.03–1.08	<0.01
MELD-Na score	1.06	1.04–1.09	<0.01
ALBI score	2.25	1.66–3.07	<0.01
ALBI grade	2.27	1.67–3.10	<0.01
Hepatic hydrothorax	1.37	1.00–1.89	0.05

**Table 3:** Multivariate Cox proportional hazards model in the entire matched cohort (*n* = 194 patients).

Variables	Hazard Ratio	95% Confidence Interval	*p*-Value
MELD-Na score	1.03	1.00–1.06	0.04
ALBI grade	1.66	1.15–2.38	0.01
Hepato-renal syndrome during hospital stay	2.60	1.05–6.41	0.03
Grade III ascites	1.58	1.13–2.21	0.01
Hepatic hydrothorax	1.18	1.00–1.68	0.04

**Table 4:** Univariate Cox proportional hazards model in the hepatic hydrothorax group.

Variable	Hazard Ratio	95% Confidence Interval	*p*-Value
Age (years)	1.01	0.99–1.04	0.14
Gender (female)	0.68	0.43–1.07	0.09
Total bilirubin (mg/dL)	1.05	1.02–1.09	<0.01
INR on admission	1.3	0.83–2.04	0.23
Creatinine on admission (mg/dL)	0.99	0.79–1.23	0.94
Kidney dysfunction during hospital stay	2.58	1.28–5.20	<0.01
Hepato-renal syndrome during hospital stay	3.91	1.40–10.87	<0.01
Sodium levels (mEq/L)	0.93	0.90–0.96	<0.01
Serum albumin (g/dL)	0.55	0.35–0.87	0.01
Grade III ascites	1.75	1.12–2.73	0.01
Spontaneous bacterial peritonitis	2.1	1.24–3.53	<0.01
Hepatic encephalopathy	1.29	0.77–2.16	0.32
Child–Pugh Class C	1.2	0.77–1.86	0.4
MELD score	1.05	1.01–1.09	<0.01
MELD-Na score	1.06	1.03–1.09	<0.01
ALBI score	2.01	1.31–3.09	<0.01
ALBI grade	1.9	1.22–2.96	0.04

**Table 5:** Multivariate Cox proportional hazards model in the hepatic hydrothorax group.

	Hazard Ratio	95% Confidence Interval	*p*-Value
MELD-Na score	1.03	1.01–1.06	0.01
ALBI grade	1.69	1.19–2.40	<0.01
Hepato-renal syndrome during hospital stay	2.43	1.31–4.51	<0.01
Grade III ascites	1.53	1.09–2.13	0.01

## Data Availability

Not applicable.
